# Supplementary material for: ChatGPT as a Tool for Biostatisticians: A Tutorial on Applications, Opportunities, and Limitations
Source: Stat Med. 2025 Oct 23;44(23-24):e70263. doi: 10.1002/sim.70263 (PMC12548020; doi:10.1002/sim.70263)
Supplement: Supplementary file 1 — Data S1. Supporting Information. [file SIM-44-0-s001.zip › Supplementary Materials Additional.zip!/3.2_Diagnostic_Test/3.2_Diagnostic_Chat4.pdf]

Latent Class Analysis LCA

I would like to determine sensitivity and specificity of two diagnostic tests. I do have the diagnostic test outcomes, but I do not know the gold standard. What can I do?

Please, tell me more about the latent class analysis.

I have two populations with two different disease prevalences. I have two diagnostic tests which are conditional independent. I would like to get the log-likelihood of this setting.

What is the Hui-Walter paradigm?

Excellent questions — you are touching upon a very rich and practical area of diagnostic test evaluation when no gold standard is available. Let me address each part in detail:

🟡 1️⃣ What to do when no gold standard is available?

When you don't know the true disease status (i.e., no gold standard), you can **model it as a latent variable** — the disease status is *unobserved* but influences the observed test outcomes.

A classical method is **Latent Class Analysis (LCA)**, which treats the disease status as a latent (hidden) class and models the observed test outcomes conditionally on this class.

🟡 2️⃣ What is Latent Class Analysis (LCA)?

LCA is a statistical method where:

- You assume that each subject belongs to one of a finite number of classes (here, diseased or not diseased — so 2 classes).
- The probability of observing a particular combination of test results depends on this latent class.
- You model the test results as **conditionally independent** given the latent class (important assumption).

For **two binary tests** T_1, T_2, and latent disease status $D \in \{0, 1\}$:

- Sensitivity of test j: $Se_j = P(T_j = 1 \mid D = 1)$
- Specificity of test j: $Sp_j = P(T_j = 0 \mid D = 0)$
- Prevalence $\pi = P(D = 1)$

You estimate π, Se_1, Sp_1, Se_2, Sp_2 by maximizing the likelihood of the observed test outcomes.

🟡 3️⃣ Two populations with different prevalences

This is where the **Hui-Walter paradigm** comes in!

🟡 4️⃣ What is the Hui-Walter paradigm?

Hui & Walter (1980) showed that:

- If you have **two populations** with **different disease prevalences**, and
- You apply **two conditionally independent tests**, then
- The model is identifiable — i.e., you can estimate prevalence, sensitivity, and specificity **without a gold standard**.

🟡 5️⃣ Log-Likelihood in this setting

Setup:

- Two populations $k = 1, 2$
- Each population has prevalence π_k
- Two tests T_1, T_2, binary outcomes
- Tests are conditionally independent given the latent disease status

Observed data:

You can cross-tabulate outcomes into **4 combinations** per population:

T_1	T_2	Count in Pop 1	Count in Pop 2
0	0	$n_{00}^{(1)}$	$n_{00}^{(2)}$
0	1	$n_{01}^{(1)}$	$n_{01}^{(2)}$
1	0	$n_{10}^{(1)}$	$n_{10}^{(2)}$
1	1	$n_{11}^{(1)}$	$n_{11}^{(2)}$

Model:

Let's write the **probability of each test outcome combination**, for population k:

For diseased subjects $D = 1$, probability:

$$P(T_1 = t_1, T_2 = t_2 \mid D = 1) = Se_1^{t_1}(1 - Se_1)^{1-t_1} \cdot Se_2^{t_2}(1 - Se_2)^{1-t_2}$$

For non-diseased subjects $D = 0$, probability:

$$P(T_1 = t_1, T_2 = t_2 \mid D = 0) = (1 - Sp_1)^{t_1} Sp_1^{1-t_1} \cdot (1 - Sp_2)^{t_2} Sp_2^{1-t_2}$$

Then the **marginal probability** in population k is:

$$P_k(t_1, t_2) = \pi_k \cdot P(t_1, t_2 \mid D = 1) + (1 - \pi_k) \cdot P(t_1, t_2 \mid D = 0)$$

Log-likelihood:

Now the **log-likelihood** for both populations is:

$$\ell = \sum_{k=1}^{2} \sum_{t_1=0}^{1} \sum_{t_2=0}^{1} n_{t_1 t_2}^{(k)} \cdot \log P_k(t_1, t_2)$$

🟡 Summary of Parameters to Estimate:

- π_1, π_2 — prevalences in populations 1 and 2
- Se_1, Sp_1 — sensitivity & specificity of test 1
- Se_2, Sp_2 — sensitivity & specificity of test 2

Total 6 parameters → model is **identifiable** if:

✅ **two populations**
✅ **two tests**
✅ **conditional independence**

Final Comments

👉 This is a very elegant and practical approach.
👉 It has been widely used in epidemiology, veterinary studies, serology, etc.

If you like, I can also provide:

✅ a small numerical example
✅ R / Python code to fit this model (e.g. via `optim`, `nlm`, or `poLCA` / `randomLCA`)
✅ possible extensions (e.g. modeling dependence, adding covariates)

Would you like me to show any of these next? 🚀

I would like to see a worked example and a R script that implements this model.

I would like to add confidence intervals to the estimated sensitivities, specificities, and prevalences.

I would like to run a bootstrap version for robust confidence intervals.

I would like to reparametrize my model using a logistic parametrization for the prevalences, sensitivities, and specificities.

Please update the bootstrap function too.

Could you also perform the calculation of the 95% Confidence intervals via the hessian?

Please provide a comprehensive R code for the issues discussed in this session.

Excellent — now we are moving to a very modern and thorough version of the Hui-Walter latent class model!

Let me give you a complete and **step-by-step R example**, covering everything you asked:

1️⃣ Simulated Example Data

We will simulate 2 populations with different prevalences and two conditionally independent tests.

2️⃣ Model Specification

✅ **Latent class model** with 2 tests, 2 populations
✅ **Logistic reparametrization** for π_k, Se_j, Sp_j
✅ **Log-likelihood** and **Hessian-based confidence intervals**
✅ **Bootstrap** for robust intervals

3️⃣ Full R Script

Here is a **comprehensive R script** covering everything:

```r

# Load required packages
library(numDeriv)  # for Hessian
library(MASS)      # for mvrnorm if needed in extensions

# 1️⃣ Simulate example data --------------------------------------

# True parameters (on probability scale)
true_pi <- c(0.3, 0.6)  # Prevalence in pop 1 and 2
true_Se <- c(0.85, 0.90)  # Sensitivity of test 1 and 2
true_Sp <- c(0.80, 0.75)  # Specificity of test 1 and 2

# Sample sizes
N <- c(500, 500)

# Function to simulate one population
simulate_population <- function(N, pi, Se, Sp) {
  D <- rbinom(N, 1, pi)
  T1 <- ifelse(D == 1, rbinom(N, 1, Se[1]), rbinom(N, 1, 1 - Sp[1]))
  T2 <- ifelse(D == 1, rbinom(N, 1, Se[2]), rbinom(N, 1, 1 - Sp[2]))
  data.frame(D, T1, T2)
}

# Simulate two populations
set.seed(123)
pop1 <- simulate_population(N[1], true_pi[1], true_Se, true_Sp)
pop2 <- simulate_population(N[2], true_pi[2], true_Se, true_Sp)

# Tabulate observed counts (4 cells per population)
tabulate_counts <- function(data) {
  xtabs(~ T1 + T2, data)
}

counts1 <- tabulate_counts(pop1)
counts2 <- tabulate_counts(pop2)
```

```r
print(counts1)
print(counts2)

# 2️⃣ Log-likelihood with logistic parametrization -------------------

# Logistic (logit) and inverse
logit <- function(p) log(p / (1 - p))
inv_logit <- function(x) 1 / (1 + exp(-x))

# Log-likelihood function
loglik <- function(par) {
  # Unpack parameters
  pi1 <- inv_logit(par[1])
  pi2 <- inv_logit(par[2])
  Se1 <- inv_logit(par[3])
  Sp1 <- inv_logit(par[4])
  Se2 <- inv_logit(par[5])
  Sp2 <- inv_logit(par[6])

  # Loop over populations
  ll <- 0
  for (k in 1:2) {
    pi_k <- ifelse(k == 1, pi1, pi2)
    counts_k <- ifelse(k == 1, counts1, counts2)

    for (t1 in 0:1) {
      for (t2 in 0:1) {
        # Probabilities given D=1
        p11 <- (Se1^t1) * ((1 - Se1)^(1 - t1)) *
               (Se2^t2) * ((1 - Se2)^(1 - t2))
        # Probabilities given D=0
        p00 <- ((1 - Sp1)^t1) * (Sp1^(1 - t1)) *
               ((1 - Sp2)^t2) * (Sp2^(1 - t2))
        # Marginal probability
        P_obs <- pi_k * p11 + (1 - pi_k) * p00
        # Contribution to log-likelihood
        n_obs <- counts_k[t1 + 1, t2 + 1]
        ll <- ll + n_obs * log(P_obs)
      }
    }
  }
  return(-ll)  # return NEGATIVE log-likelihood for minimization
}

# 3️⃣ Fit model -----------------------------------------------------

# Initial values on logit scale
init_par <- rep(0, 6)  # corresponds to probability 0.5

# Optimization
fit <- optim(init_par, loglik, hessian = TRUE, method = "BFGS", control = list(maxit = 10000))

# 4️⃣ Extract estimates ---------------------------------------------

# MLE estimates on probability scale
est <- inv_logit(fit$par)
names(est) <- c("pi1", "pi2", "Se1", "Sp1", "Se2", "Sp2")
print(est)

# 5️⃣ CI from Hessian -----------------------------------------------

# Variance-covariance matrix
vcov_mat <- solve(fit$hessian)

# Delta method to transform to probability scale
se_logit <- sqrt(diag(vcov_mat))
ci_lower <- inv_logit(fit$par - qnorm(0.975) * se_logit)
ci_upper <- inv_logit(fit$par + qnorm(0.975) * se_logit)

# Summary table
results <- data.frame(
  Parameter = names(est),
  Estimate = est,
  CI_lower = ci_lower,
  CI_upper = ci_upper
```

```r
)
print(results)

# 6️⃣ Bootstrap -----------------------------------------------------

# Bootstrap function
bootstrap_fit <- function(B = 1000, seed = 1234) {
  set.seed(seed)
  boot_estimates <- matrix(NA, nrow = B, ncol = 6)

  for (b in 1:B) {
    # Resample each population with replacement
    pop1_boot <- pop1[sample(nrow(pop1), replace = TRUE), ]
    pop2_boot <- pop2[sample(nrow(pop2), replace = TRUE), ]

    # Recompute counts
    counts1_boot <- tabulate_counts(pop1_boot)
    counts2_boot <- tabulate_counts(pop2_boot)

    # New loglik function using local copies of counts
    loglik_boot <- function(par) {
      pi1 <- inv_logit(par[1])
      pi2 <- inv_logit(par[2])
      Se1 <- inv_logit(par[3])
      Sp1 <- inv_logit(par[4])
      Se2 <- inv_logit(par[5])
      Sp2 <- inv_logit(par[6])

      ll <- 0
      for (k in 1:2) {
        pi_k <- ifelse(k == 1, pi1, pi2)
        counts_k <- ifelse(k == 1, counts1_boot, counts2_boot)

        for (t1 in 0:1) {
          for (t2 in 0:1) {
            p11 <- (Se1^t1) * ((1 - Se1)^(1 - t1)) *
                   (Se2^t2) * ((1 - Se2)^(1 - t2))
            p00 <- ((1 - Sp1)^t1) * (Sp1^(1 - t1)) *
                   ((1 - Sp2)^t2) * (Sp2^(1 - t2))
            P_obs <- pi_k * p11 + (1 - pi_k) * p00
            n_obs <- counts_k[t1 + 1, t2 + 1]
            ll <- ll + n_obs * log(P_obs)
          }
        }
      }
      return(-ll)
    }

    # Fit bootstrap sample
    fit_boot <- optim(init_par, loglik_boot, method = "BFGS", control = list(maxit = 10000))

    # Store estimates on probability scale
    boot_estimates[b, ] <- inv_logit(fit_boot$par)

    if (b %% 100 == 0) cat("Bootstrap iteration:", b, "\n")
  }

  # Return bootstrap matrix
  return(boot_estimates)
}

# Run bootstrap
boot_results <- bootstrap_fit(B = 1000)

# Compute bootstrap percentile CIs
boot_CI <- apply(boot_results, 2, quantile, probs = c(0.025, 0.975))

# Format bootstrap summary
boot_summary <- data.frame(
  Parameter = c("pi1", "pi2", "Se1", "Sp1", "Se2", "Sp2"),
  Estimate = est,
  Boot_CI_lower = boot_CI[1, ],
  Boot_CI_upper = boot_CI[2, ]
)
```

```
print(boot_summary)

# Done! -----------------------------------------------------------
```

🚀 What this script gives you:

✅ Simulated example of two populations and two tests
✅ Full likelihood with **logistic parametrization**
✅ **Optimization** using `optim`
✅ **Hessian-based confidence intervals**
✅ **Bootstrap CIs**
✅ Modular code (easy to adapt to your own data!)

📌 Summary

You now have a **general template** for:

1️⃣ **Latent Class Model with 2 tests, 2 populations**
2️⃣ **Logistic parametrization** of all probabilities
3️⃣ **Likelihood maximization**
4️⃣ **Hessian-based intervals**
5️⃣ **Bootstrap intervals**

Would you like me to also show:

✅ How to extend this to **more than 2 populations**
✅ How to handle **conditional dependence** (e.g. using covariance terms)
✅ How to make a **nice publication-quality table** of the results

Just tell me how far you want to go — this is a powerful framework! 🚀
